# Annotations

**Published:** 1887-08-20

**Authors:** 


### Annotations.

201 students were capped at the Edinburgh University Graduation ceremonial the other day, each of them now being fully licensed to take charge of the health of her Majesty's lieges. These do not represent a quarter of the young doctors who are annually let loose upon the inhabitants of the British Isles to cure or to kill as the case may be. Over a thousand fairly capable men are yearly withdrawn from productive pursuits to bolster up the tottering strength of the community. A sad consideration for the community that such an army should be needed; and a still more melancholy reflection for the doctors that their lives should be spent in a career in which their greater success represents the greater trouble and sorrow of their unhappy clients. Who pities the doctor in that he can only be prosperous as the result of other people's griefs? Many people regard him as a greedy man who sticks to the house like a leech when once he has been called in. Even if it were so it is not to be wondered at, when it is considered how few are the patients which fall to many a medical man's lot; how much fewer still they are who pay him his just dues, and how vast is the army of invading competitors annually poured into the ranks of medicine by the different medical schools. The British parent does not look very far ahead, or he would think twice before sending his son into a profession where to many men the chief rewards are, excessive labour, increasing anxiety, and a minimum of pecuniary reward.

**A Deluge of Doctors.**

**Men must do Something!**

It is argued that young men must do something. Most true: they must either work or starve. In the present shifting condition of theological opinion and ecclesiastical establishments the Church is not looked upon as offering a very safe career. The Bar presents a still more disheartening spectacle than the medical profession. Commerce is so over-run by men of all classes and nationalities, that reflecting persons hesitate before they venture their barque upon such a surging sea. What remains? The land, and the occupations requiring the wearing of rough clothing, and the bidding good-bye to the outer aspects of gentility. Is the land supporting all the people it is capable of supporting? Certainly not. No doubt alterations in the conditions of tenure are necessary and imminent, but there is still greater necessity for change in the character and methods of those who cultivate our fields. Farmers, as a class, are behind the times. They have been too long accustomed to a life of semi-laziness for six or eight months in the year, to be able and willing to put forward that skill and energy which the pressure of the period requires. Many young men who now become doctors, if they would devote the same labour and thought to agricultural pursuits which they give to the acquisition of medical knowledge and practice, might earn three times as much money as the average practitioner, and at the same time have a life incomparably more healthy and happy. The land is becoming more and more deserted, to the great injury of the community and the increasing pressure on other callings. Where is the statesman who shall restore the people to the healthiest of all callings?

**Why don't Doctors emigrate?**

Doctors do emigrate, and that in ever-increasing numbers. Any man who will take ship and circumnavigate the globe, after the fashion of Captain Cook and later explorers, will find it difficult to get beyond the reach of English, and especially Scotch doctors. There is hardly a city of any importance in Europe, Asia, Africa, or America, where a doctor speaking the Saxon tongue may not be found. In Australia and other colonies they are almost as abundant as the descendants of the rabbits sent out from this country, and threaten to become as great a nuisance if they continue to increase. The men of commerce are always crying out for new markets to be opened up in different parts of the globe, and doctors are beginning to put in their claims for "fresh fields and pastures new." Our old Norse forefathers would have promptly settled the question by preliminary prospecting and a subsequent invasion, driving out or subordinating the native populations. Is there no quarter of the globe where an army of doctors might, by a little previous killing, establish themselves securely, either as proprietors, cultivators, or medicine-men? It is worth a thought by the younger scions of physic.

**A Short Way with Medical Aspirants.**

Malthus and other similar reasoners have always been fond of short cuts. The undisciplined and the highly cultivated intellect agree in liking all manner of abbreviations, and hence we find that Eastern monarchs do not as a rule allow practical questions to interfere with the soundness of their slumbers. Heads may be cut off as well as counted in a thoroughly well-regulated state. Unhappily our own aristocratico-democratic state is by no means well regulated, and there is a weak and unstatesmanlike objection to the very convenient method of decapitation. There is no likelihood of diminishing the number of doctors by consent of Parliament, and we must therefore hope to accomplish our object by the consent of Paterfamilias. Let him but once be thoroughly convinced that the game is by no means worth the candle, and we shall soon have a much diminished keenness of competition in the medical market. When the rooks and other birds desire to share the farmer's corn, of which the supply is altogether insufficient for himself, he employs a boy or boys to parade the field with an alarming rattle to drive the intruders away. Could not the doctors, who see starvation coming upon them at no distant period, invent a rattle of their own, whereby foolish birds might be induced, at any rate for a time, to leave them and their crops in peace?

